# Cost‐effectiveness of prophylactic hysterectomy in first‐degree female relatives with Lynch syndrome of patients diagnosed with colorectal cancer in the United States: a microsimulation study

**DOI:** 10.1002/cam4.4080

**Published:** 2021-09-12

**Authors:** Maaike Alblas, Elisabeth F. P. Peterse, Mengmeng Du, Ann G. Zauber, Ewout W. Steyerberg, Nikki van Leeuwen, Iris Lansdorp‐Vogelaar

**Affiliations:** ^1^ Department of Public Health Erasmus MC–University Medical Center Rotterdam Rotterdam The Netherlands; ^2^ Public Health Sciences Division Fred Hutchinson Cancer Research Center Seattle WA USA; ^3^ Department of Epidemiology & Biostatistics Memorial Sloan Kettering Cancer Center New York NY USA; ^4^ Department of Biomedical Data Sciences Leiden University Medical Center Leiden The Netherlands

**Keywords:** advisory committees, cost‐effectiveness, hysterectomy, lynch syndrome, microsimulation, microsimulation model, theoretical

## Abstract

**Background:**

To evaluate the cost‐effectiveness of prophylactic hysterectomy (PH) in women with Lynch syndrome (LS).

**Methods:**

We developed a microsimulation model incorporating the natural history for the development of hyperplasia with and without atypia into endometrial cancer (EC) based on the MISCAN‐framework. We simulated women identified as first‐degree relatives (FDR) with LS of colorectal cancer patients after universal testing for LS. We estimated costs and benefits of offering this cohort PH, accounting for reduced quality of life after PH and for having EC. Three minimum ages (30/35/40) and three maximum ages (70/75/80) were compared to no PH.

**Results:**

In the absence of PH, the estimated number of EC cases was 300 per 1,000 women with LS. Total associated costs for treatment of EC were $5.9 million. Offering PH to FDRs aged 40–80 years was considered optimal. This strategy reduced the number of endometrial cancer cases to 5.4 (−98%), resulting in 516 quality‐adjusted life years (QALY) gained and increasing the costs (treatment of endometrial cancer and PH) to $15.0 million (+154%) per 1,000 women. PH from earlier ages was more costly and resulted in fewer QALYs, although this finding was sensitive to disutility for PH.

**Conclusions:**

Offering PH to 40‐ to 80‐year‐old women with LS is expected to add 0.5 QALY per person at acceptable costs. Women may decide to have PH at a younger age, depending on their individual disutility for PH and premature menopause.

## Graphical abstract

We evaluated the cost‐effectiveness of prophylactic hysterectomy (PH) in women with Lynch syndrome (LS). Offering PH to 40–80 year old women with LS is expected to add 0.5 QALY per person at acceptable costs. Women may decide to have PH at age 35 years, depending on their individual disutility for PH and premature menopause.

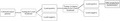

## 1 INTRODUCTION

It has been standard policy for years to try and identify Lynch Syndrome (LS) mutation carriers among colorectal cancer (CRC) patients. Initially, this was done using family history criteria, but since the past decade, universal reflex testing of tumors of CRC patients for mismatch repair deficiency has become increasingly accepted. The aim of this practice is to identify first‐degree relatives (FDR) with LS, in order to provide them with preventive interventions.^1^, ^2^, ^3^, ^4^, ^5^ LS is a hereditary condition that causes a substantial risk of both colorectal cancer (30%–60%) and endometrial cancer (17%–60%).^6^, ^7^, ^8^, ^9^ It is estimated that approximately 1 in 300 individuals have LS in the United States (US). ^10^, ^11^, ^12^ The practice of universal testing for LS and offering FDR with LS intensive colonoscopy screening for colorectal cancer has shown to be (cost‐)effective.^13^, ^14^ Yearly endometrial sampling from age 30–35 years onwards might be considered a possible screening strategy for female carriers, but there is no consensus on the effectiveness and impact on quality of life of this strategy.^15^ Prophylactic hysterectomy combined with oophorectomy (further referred to as prophylactic hysterectomy, PH) when childbearing is completed has been suggested as a preventive strategy.^4^, ^16^ It might prevent nearly all endometrial cancer cases and deaths in women with LS.^4^, ^16^ However, little is known about its cost‐effectiveness and the optimal age range. Determining this optimal age range requires to consider different elements that are associated with PH, such as costs and quality of life. One study using a Markov model showed that offering prophylactic hysterectomy from age 40 is cost‐effective, but these results were based on a single‐age cohort and only a limited number of strategies (two minimum ages and no maximum age).^17^ In reality, the age distribution of identified LS carriers ranges from 11 to 80.^18^ This age range is of specific importance because women at higher ages should be able to weigh the benefits and harms of surgery, given that they have not developed symptomatic endometrial cancer. To our knowledge, no previous study has incorporated the age range of LS carriers in their modelling. The aim of this study was to evaluate the cost‐effectiveness of offering prophylactic hysterectomy to female FDR with LS, comparing different age ranges to assess optimal age thresholds. Therefore, we developed a microsimulation model for endometrial cancer based on the MISCAN modeling framework.

## 2 METHODS

### 2.1 Model specification and assumptions

We used the well‐established MISCAN model as a framework to develop the MISCAN Endometrial model. The MISCAN model has been extensively described elsewhere.^19^, ^20^ In short, the MISCAN models simulate a large population of individuals, including life histories from birth to death. The simulations are based on input parameters, which contain both demographic information and the natural history of the specific disease. The results of the MISCAN models include information on age‐specific disease incidence and mortality.

**FIGURE 1:**
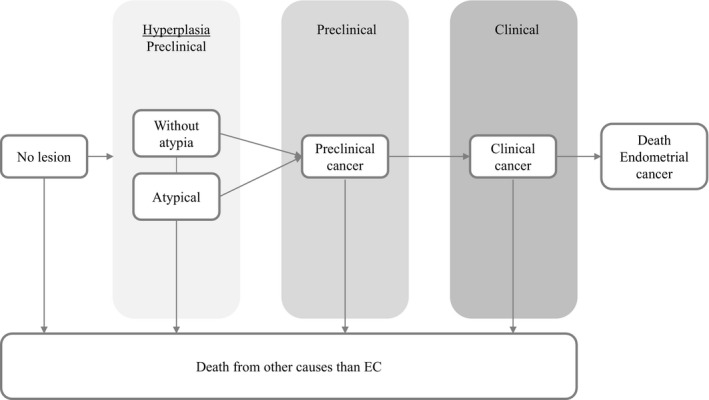
Natural history model of MISCAN Endometrium model. EC, endometrial cancer

The natural history part of the model is shown in Figure 1 and divides the development of endometrial cancer in three sequential phases: preclinical hyperplasia, preclinical cancer, and clinical cancer.^8^ We assumed two types of hyperplasia, of which endometrial hyperplasia without atypia is 6.14 times more frequent than atypical endometrial hyperplasia.^21^ The progression of hyperplasia to endometrial cancer differed between hyperplasia without atypia and hyperplasia with atypia, since both have different dwelling times.^21^ Dwelling times were derived from Lacey et al. and were estimated with a Weibull distribution.^21^ In line with assumptions made for the development of colorectal cancer,^14^ preclinical lesions were assumed to progress 10 times faster in LS patients than in the general population. The age‐specific onset of endometrial hyperplasia was calibrated to match the incidence of EC for LS women according to Bonadona et al.^8^ The survival rates were based on SEER 18 data and were corrected for death due to other causes.^22^ Upon of diagnosis of EC, death can occur due to EC or other causes. An elaborative description of our MISCAN model can be found in the Supporting Information Model Appendix.

### 2.2 Study population

**FIGURE 2:**
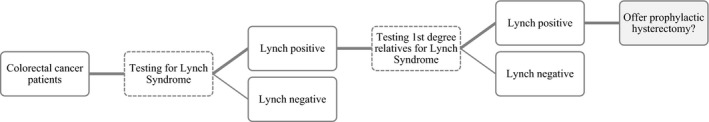
Flowchart target population for prophylactic hysterectomy

For each EC prevention strategy, we simulated a population of 10 million Lynch positive women. The target population for prophylactic hysterectomy consisted of FDR with LS of colorectal cancer patients with LS (Figure 2). The age range of the population simulated matched that of FDR with LS in a Dutch study of universal testing of LS in colorectal cancer.^18^ Individuals were between age 11 and 80 when they were diagnosed with LS. Their median age was 42 years, with an interquartile range of 31–55 years. In addition, benefits and costs of PH by 5‐year age groups were computed.

### 2.3 Strategies

Nine different age ranges were modeled with varying ages at which prophylactic hysterectomy was offered as young as 30, 35, or 40 years and as old as age 70, 75, or 80 years. Prophylactic hysterectomy was considered to eliminate the risk of EC completely from the date of surgery. We assumed full compliance of every woman who was invited for prophylactic hysterectomy.

### 2.4 Data and assumptions for costs and utilities

An overview of the costs and utilities that were used in the model can be found in Table 1. We assumed that prophylactic hysterectomy reduced the quality of life because of surgically induced menopause. The first month after surgery, quality of life was valued at 0.56, followed by 0.74 in the second and third month after surgery.^23^, ^24^, ^25^, ^26^ From three months onward, we assumed a utility of 0.88 and corrected the quality of life up to the age of 45, as it is assumed that natural menopause starts at this age which eliminates the negative side effects on quality of life of prophylactic hysterectomy.^17^, ^24^, ^27^ We also adjusted the quality of life of women diagnosed with EC.^17^, ^28^ The costs of prophylactic hysterectomy are reported as total Medicare reimbursement and include gynecologist fee, anesthesia fee for hysterectomy, pathology fee for uterus, inpatient diagnosis‐related group fees, and preoperative lab fees.^29^ For the costs of treatment of EC, we assumed 25% of all LS patients receive radiotherapy and 15% of LS patients receive chemotherapy.^16^, ^30^ Furthermore, we included gynecologist fee, anesthesia fee for hysterectomy, pathology fee for uterus, inpatient diagnosis‐related group fees, pathology fee for lymph nodes and preoperative lab fees.^29^

**TABLE 1:** Model inputs

Variable	Base case	Range	Reference
Cumulative risk of developing EC before age 80	35%	17–60	Bonadona 2011^8^
Age distribution of FDR^a^	11–80	—	Leenen 2016^18^
Survival probability	Age specific	—	SEER 2009–2013
Ratio of prevalence of hyperplasia without atypia compared to with atypia	6.14	—	Lacey 2010^21^
Life table	Age specific	—	National Vital Statistics Reports 2012^44^
Dwelling time atypical lesions	7.77		Assumption^b^
Dwelling time lesions without atypia	114.40		Assumption^b^
Costs prophylactic hysterectomy^c^	15,276	7,638–30,552	Havrilesky 2009^29^
Costs EC^d^	35,763	17,882–71,526	Schmeler 2006^16^ Broaddus 2006^30^
Utility prophylactic hysterectomy	0.88	0.82–0.99	Roberts 2011^23^ Bhattacharya 2011^25^ Hurskainen 2004^26^
Utility well	1	0.8–1.0	Fryback 1993^45^

Abbreviations: EC, endometrial cancer; FDRs, first‐degree relatives.

^a^ The median age was 42 years, with an interquartile range of 31–55 years.

^b^ We derived dwelling times from Lacey et al. (2010) with a Weibull distribution. We assumed that for women with Lynch Syndrome, dwelling times were 10 times shorter as for the general population. Values are shown as mean input parameter, dwelling times of lesions that develop into EC will be shorter.^46^

^c^ Cost reported as total Medicare reimbursement in US dollars. Includes: gynecologist fee, anesthesia fee for hysterectomy, pathology fee for uterus, inpatient diagnosis‐related group fees, preoperative lab fees.

^d^ For the costs of treatment of EC, we assumed 25% of all LS patients receive radiotherapy and 15% of LS patients receive chemotherapy^16^, ^30^

### 2.5 Outcomes

We determined the effects of offering prophylactic hysterectomy in terms of number of EC deaths, number of prophylactic hysterectomies, life years gained (LYG) and quality‐adjusted life years gained (QALYG). We calculated the associated costs for each strategy based on number of prophylactic hysterectomies and total treatment costs for endometrial cancer. We applied a 3% discount rate for both effects and costs to the year in which the women were diagnosed with LS, except for the number of EC cases and deaths. Our analyses were performed with the assumptions described in Table 1. We evaluated average cost‐effectiveness ratios (ACERs), which are defined as the difference in costs divided by the difference in QALYG compared to the no prophylactic hysterectomy strategy. Next, the incremental cost‐effectiveness ratios (ICERs) of the different strategies were evaluated to determine the optimal strategy. We assumed a willingness‐to‐pay threshold of 100,000 US dollars per QALY for this analysis.^31^, ^32^

### 2.6 Sensitivity analyses

To evaluate which assumptions were important drivers for our conclusion, we performed several sensitivity analyses (see range in Table 1). We varied: (1) Quality of life of endometrial cancer, prophylactic hysterectomy and health state well; (2) costs of (prophylactic) hysterectomy; (3) risk of endometrial cancer; and (4) lower life expectancy due to colorectal cancer risk in LS.

## 3 RESULTS

In the absence of prophylactic hysterectomy in FDRs with LS, the MISCAN‐Endometrium model predicted 300 EC cases and 71 EC deaths per 1,000 women with LS, accounting for the age distribution of the FDR at LS diagnosis. Total associated costs for the treatment of EC were estimated at $5.9 million. Offering these women prophylactic hysterectomy greatly reduced the number of EC cases and deaths, ranging from 0 to 11 and of 0 to 2.9 per 1,000 women, respectively. Although the number of LYG varied relatively little between the different strategies (411–435 per 1,000 women), the number of QALYG was substantially higher for strategies with age 40 as a start age (506–516 per 1,000 women) compared to age 35 (374–384 per 1,000 women) and age 30 (262–272). All strategies with prophylactic hysterectomy were cost‐effective compared to no prophylactic hysterectomy, with ACERs below $50,000 when either LYG or QALYG were used as effectiveness measures (Table 2).

**TABLE 2:** Results per 1000 women diagnosed with Lynch syndrome

Strategy	EC cases	EC deaths	LYG^a^, ^b^	QALYG^a^, ^b^	Costs^a^, (million US$)	ACER QALYG^a^, ^b^
No prophylactic hysterectomy	300	70.9	**—**	**—**	5.9	
30–70	5.6	2.0	426	262	14.1	$31,220
30–75	1.3	0.5	433	269	14.4	$31,618
30–80	0.0	0.0	435	272	14.6	$31,936
35–70	6.6	2.1	423	374	13.7	$20,735
35–75	2.3	2.9	430	381	14.0	$21,228
35–80	1.0	0.2	432	384	14.2	$21,513
40–70	11.0	2.9	411	506	13.2	$14,306
40–75	6.7	1.5	417	514	13.5	$14,768
40–80	5.4	1.0	420	516	13.7	$15,008

Abbreviations: ACER, Average Cost‐Effectiveness Ratio; EC, deaths endometrial cancer deaths; LYG, life years gained; QALYG, quality‐adjusted life years gained.

^a^ Results are 3% discounted.

^b^ Compared to no prophylactic hysterectomy.

**FIGURE 3:**
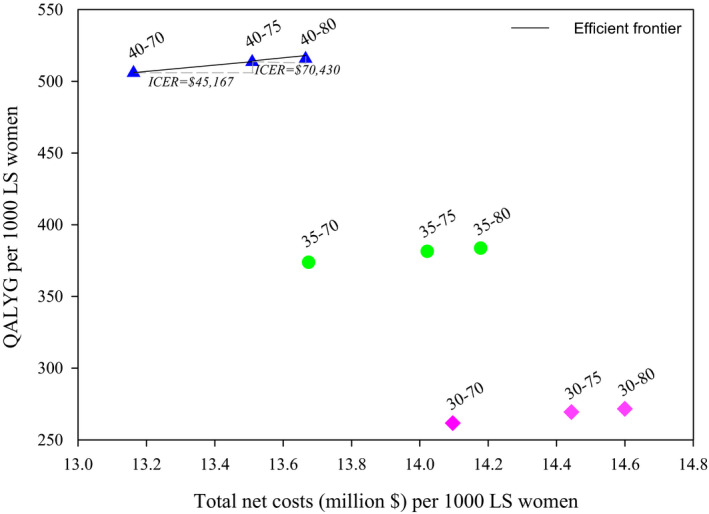
Efficiency frontier quality‐adjusted life years gained. QALYG, quality‐adjusted life‐years gained, LS, Lynch syndrome

When adjusting for quality of life, only strategies in which prophylactic hysterectomy was offered to FDRs after age 40 were efficient strategies; strategies that included prophylactic hysterectomy from age 30 and age 35 were more costly and resulted in fewer quality‐adjusted life years gained (Figure 3). The ICERs for ages 40–75 and ages 40–80 were $45,167 and $70,430, respectively. Assuming a willingness‐to‐pay threshold of $100,000, offering prophylactic hysterectomy to LS women aged 40–80 was considered optimal. Compared to no prophylactic hysterectomy, this strategy would reduce the number of endometrial cancer cases to 5.4 (−98%), resulting in 516 quality‐adjusted life years gained and increasing the costs (treatment of endometrial cancer and prophylactic hysterectomy) to $15.0 million (+154%) per 1,000 women. That PH before age 40 is not cost‐effective can easily be seen from Table 3. For example, offering PH to women aged 30–34 prevents 77.9 EC deaths compared to 76.2 EC deaths for PH, women aged 40–44 prevents (Table 3), which is an increase of 2.2%. The life‐years with PH before age 45 on the other hand increase from approximately 2.5 years to 12.5 years, an increase of 400%. At the other extreme, Table 3 also clearly outlines why PH is still worthwhile even up to age 80: in 75–79 year‐olds still more than 40 EC deaths per 1,000 women can be prevented, while the disutility from PH at that age is small, because we only assume disutility in the first three months after surgery.

**TABLE 3:** Results per age category (per 1000 women diagnosed with Lynch syndrome)

Strategy	EC cases prevented	EC deaths prevented	LYG^a^, ^b^	QALYG^a^	Additional Costs^a^ (million US$)
30–34	351.8	77.9	460	−489	9.518
35–39	348.5	77.6	510	45	8.811
40–44	339.5	76.2	536	608	8.269
45–49	323.4	73.8	534	918	7.975
50–54	297.1	70.3	502	845	8.087
55–59	258.1	65.8	443	701	8.754
60–64	217.8	60.9	385	558	9.544
65–69	178.8	55.0	320	420	10.372
70–74	142.2	48.0	252	292	11.210
75–79	108.8	40.7	188	182	12.033

Abbreviations: EC, deaths endometrial cancer deaths; LYG, life years gained; QALYG, quality‐adjusted life years gained.

^a^ Results are 3% discounted.

^b^ Earlier PH adds slightly more LYG for women who would otherwise die from EC between this age group and the next. On the other hand, LYG in all women who would be diagnosed with EC after age 35 are discounted for 5 more years and therefore become smaller.

### 3.1 Sensitivity analyses

The findings of this study were robust for most of our assumptions (Supporting Information Appendix Table 5–14). Only when a higher utility after PH was assumed or life‐years gained were considered as the primary outcome, offering prophylactic hysterectomy before age 40 was optimal. However, there were no model‐recommended strategies with starting ages below 35 years. The recommended stop age was age 80 in all analyses, except when higher hysterectomy costs were assumed (Table 4).

**TABLE 4:** Model‐recommended strategies with a willingness‐to‐pay threshold of $100,000 based on varying input parameters in sensitivity analyses

	Model recommended strategies
Base case	40–80
Base case without adjustment for quality of life	30–80
Prophylactic hysterectomy costs	
−50%	40–80
+100%	40–75
Treatment costs endometrial cancer	
−50%	40–80
+100%	40–80
Utility endometrial cancer	
0.68	40–80
Utility prophylactic hysterectomy	
0.82	40–80
0.99	35–80
Risk of endometrial cancer	
17%	40–80
60%	40–80
Accounting for reduced life expectancy due to increased colorectal cancer risk in LS^a^	40–80

Abbreviations: LYG, life years gained; QALYG, quality‐adjusted life‐years gained.

^a^ MISCAN‐Colon was used to generate lifetables that accounted for the increased colorectal cancer mortality of LS women, assuming LS women participated in biennial colonoscopy surveillance from age 25 to age 80.^14^

## 4 DISCUSSION

We evaluated the cost‐effectiveness of offering prophylactic hysterectomy to asymptomatic women diagnosed with LS by reflex testing and subsequent cascade testing of FDR with colorectal cancer. Our results show that offering prophylactic hysterectomy to these women is cost‐effective at currently accepted standards, and is most cost‐effective when offered between age 40 and 80. Depending on an individual disutility for PH and premature menopause, women may decide to undergo PH at a younger age when the perceived impact of PH and premature menopause is small.

Obviously, earlier stop ages were optimal when higher costs of hysterectomy were assumed. The increase in benefits of offering prophylactic hysterectomy to LS women until age 80 rather than age 70 or 75 was relatively small. This may be explained by the median age of diagnosis of endometrial cancer in patients with LS, which is 48 years,^33^ while 98% may be diagnosed before the age of 65 years.^33^ This may support stopping prophylactic hysterectomy before age 70 to prevent potential unnecessary surgery. However, as long as the relative increase in costs is also small, offering prophylactic hysterectomy until age 80 may be considered.

Altering the input parameters for quality of life after PH resulted in the recommendation to start prophylactic hysterectomy at an age younger than 40 years. Women will go into premature menopause as a result of prophylactic hysterectomy, which can result in depression, anxiety, sexual dysfunction and lower self‐confidence.^34^ We must acknowledge the presence of individual variation in the impact of PH on quality of life during premature menopause. Little is known on this individual variation and specific data on utilities after prophylactic surgery instead of curative surgery is currently lacking. Therefore, empirical data regarding quality of life after prophylactic hysterectomy and the resulting premature menopause are needed to make the quality of life adjustments that are made in our model more robust.

An important strength of this study is that it comprehensively compares the cost‐effectiveness of offering prophylactic hysterectomy to women diagnosed with LS for different minimum and maximum ages in a mixed population of different ages. Our results are in line with the results from a prior Markov decision model by Kwon et al,^17^ who also showed that offering prophylactic hysterectomy from age 40 was the best strategy. Like us, Kwon et al^17^ also showed that the results are highly depended on the inclusion of quality of life in the analyses. In our analyses, starting with prophylactic hysterectomy at age 30 until age 80 prevented all endometrial cancer cases and deaths due to endometrial cancer, leading to a high number of LYG. However, this strategy comes at a high prize in terms of costs and quality of life. Hence, any strategy that starts at the age of 30 or even age 35 was dominated by strategies that start prophylactic hysterectomy at age 40. In addition, the age when women have their first child is increasing, which might cause women to complete their family at an older age.^35^ As a consequence, women may postpone prophylactic hysterectomy. Yang et al^36^ identified prophylactic hysterectomy from age 30 as optimal strategy, compared to annual examination. However, no other start ages were tested, which complicates the comparison with the results from our study.

Furthermore, the results of our study are applicable to all asymptomatic women with LS. Although the target population of our study consisted of FDR with LS of colorectal cancer patients with LS, the target population might also be FDR of patients diagnosed with EC. However, the majority of asymptomatic LS patients is identified through a colorectal cancer case in the family, which was therefore the focus of our current analysis. The only parameter in the model that was influenced by this assumption is the age distribution of the asymptomatic LS cases, which was only available for those related to a colorectal cancer patient. As the median ages of colorectal cancer and endometrial cancer diagnoses are comparable, the age distribution of first‐degree relatives identified with LS are likely also comparable. Therefore, the results of our study are applicable to all asymptomatic women with LS, regardless of whether they were related to a colorectal cancer or an endometrial cancer patient.

Some limitations of our study should be acknowledged. First, we used the utilities and costs of hysterectomy combined with oophorectomy in our analyses, while we did not incorporate ovarian cancer in our microsimulation model. We have chosen to do so because prophylactic hysterectomy combined with oophorectomy has been recommended as preventive strategy in female patients with LS, given their elevated risk of ovarian cancer (2%–39% life time risk).^16^ However, recent studies have shown that ovarian cancer is often detected at an early stage in LS patients, with a relatively good 10‐year survival prognosis of 81%.^37^, ^38^, ^39^ Hence, it might be an option to offer younger women the option to undergo a single prophylactic hysterectomy as initial surgery, and to undergo a delayed bilateral salpingo‐oophorectomy at menopause. This two‐step surgery option might influence the decision of women to undergo prophylactic surgery, since this option does not result in premature menopause. Given the changes in costs and quality of life, some effect on the cost‐effectiveness is expected. Based on our sensitivity analysis, in which we assumed a higher utility after prophylactic hysterectomy, we expect that a younger starting age for prophylactic hysterectomy will be the optimal strategy. Future studies are necessary to determine if treatment options such as prophylactic hysterectomy with delayed bilateral salpingo‐oophorectomy at menopause are (1) safe for LS patients given their elevated risk of ovarian cancer, and (2) cost‐effective.

Second, we assumed that every woman who was invited for prophylactic hysterectomy would undergo this procedure. The model therefore predicted the maximum achievable benefits of prophylactic hysterectomy. Although this implies that the predicted benefits are unrealistic, guidelines should be made based on the benefits that would accrue under perfect rates of adherence to recommendations. Moreover, any change in rates of adherence will have no effects on the ratios that were calculated in our analyses, as the costs and benefits that were used are proportional. Research has shown that FDR of patients with LS underutilize genetic screening, with uptake varying from 15% to 53%.^40^ A study on the uptake of bilateral risk‐reducing mastectomy and bilateral risk‐reducing salpingo‐oophorectomy amongst BRCA1/2 mutation carriers showed that uptake was 40% and 45% respectively, and was related to lifetime risk and age.^41^ Third, we did not consider other LS‐related cancers, such as colorectal or ovarian cancer; due to the lack of data we assumed that apart from an increased EC risk, LS cases have a normal life expectancy. This potentially resulted in an overestimation of life‐years gained per EC death prevented. However, our sensitivity analysis showed that our findings were robust when we corrected life expectancy for the increased colorectal cancer mortality in LS. Fourth, the natural history of EC in women with LS is largely unknown. In line with analyses performed for colorectal cancer in LS, we assumed that dwelling times are ten times shorter for women with LS compared to the general population. Fifth, the risk of EC in LS women is uncertain, as estimates vary greatly among studies.^8^ We calibrated our model to the largest study that accounted for ascertainment bias,^6^, ^7^, ^8^ and explored higher and lower risk levels in sensitivity analyses. Our results demonstrate that the optimal age range depends on the assumed EC risk for LS cases, which is why future studies are needed to determine the exact risk of EC in LS women. Lastly, we assumed Medicare costs in our analysis while most women might not be Medicare eligible. Also we do not account for non‐medical costs such as out‐of‐pocket costs or time out of work. The current costs might therefore be an underestimation of the costs associated with PH and the treatment of EC. Furthermore, we were unable to find recent cost data to use in our analyses, which might also contribute to an underestimation of the costs as we assumed that the somewhat older cost data were applicable to recent practice. Further studies are necessary to determine these type of costs to enrich existing cost‐effectiveness analyses. Nonetheless, sensitivity analysis found our conclusions to be robust for our assumptions on costs and this underestimation will likely not have influenced our conclusions. We did not perform a probabilistic sensitivity analysis since it is not feasible to provide reliable confidence intervals around our estimates due to the lack of data on the distribution of most of the parameters Therefore, we have chosen to conduct several one‐way sensitivity analyses. The results of these sensitivity analyses indicate that the findings of our study were robust for most of our assumptions.

Current guidelines in the United States recommend to offer prophylactic hysterectomy to women from age 40 or when childbearing has completed.^3^ This is in line with the results from our study and underlines the importance of identifying LS mutation carriers among colorectal cancer patients and subsequent cascade testing to improve future prospects of these patients in terms of life expectancy and quality of life. However, standards and protocols vary between centers and countries, which may lead to undesired variation.^42^ This variation may be caused by conflicting recommendations and protocols on the optimal screening and preventive strategy for LS.^43^ Additional information regarding costs and effects of prophylactic hysterectomy, as provided by our study, may aid in the development of uniform protocols and recommendations for the identification of LS mutation carriers. Moreover, our results can inform physicians and women with LS regarding the decision whether or not to perform prophylactic hysterectomy and from which age, which is important in determining the optimal strategy given the preference‐sensitive nature of the decisions these patients are facing.

In summary, our study suggests that offering prophylactic hysterectomy to women diagnosed with LS is cost‐effective, and is most cost‐effective when offered from age 40 until age 80. Individual variation in impact of PH and premature menopause on quality of life must be taken into account and may cause women to start PH earlier. These findings can be used to inform policy makers and clinicians regarding decisions about offering prophylactic hysterectomy to LS women.

## CONFLICTS OF INTEREST

The authors declare that there are no conflicts of interest.

## AUTHOR CONTRIBUTIONS

Maaike Alblas: data curation, formal analysis, methodology, writing ‐ original draft, writing ‐ review and editing. Elisabeth F.P. Peterse: data curation, formal analysis, methodology, writing ‐ original draft, writing ‐ review and editing. Mengmeng Du: data curation, writing ‐ review and editing. Ann G. Zauber^:^ data curation, writing ‐ review and editing. Ewout W. Steyerberg: data curation, supervision, writing ‐ review and editing. Nikki van Leeuwen: methodology, supervision, writing review and editing. Iris Lansdorp‐Vogelaar: conceptualization, methodology, supervision, data curation, writing ‐ original draft, writing ‐ review and editing.

## ETHICAL APPROVAL

No ethical approval was sought prior to commencing this study, as this study only contains simulated data.

## Supporting information

Supplementary Material

AppendixTables5‐14

## Data Availability

The data that support the findings of this study are available from the corresponding author upon reasonable request.
