# Supplementary material for: Cost‐effectiveness of prophylactic hysterectomy in first‐degree female relatives with Lynch syndrome of patients diagnosed with colorectal cancer in the United States: a microsimulation study
Source: Cancer Med. 2021 Sep 12;10(19):6835–44. doi: 10.1002/cam4.4080 (PMC8495276; doi:10.1002/cam4.4080)
Supplement: Supplementary file 1 — Supplementary Material [file CAM4-10-6835-s002.docx]

Model Appendix

**Microsimulation model structure**

The Microsimulation Screening Analysis (MISCAN) program was first developed in 1985 to evaluate the effects of screening on disease.^1^ Since then, the MISCAN program has been used to quantify the effects of primary and secondary prevention for cancers of the breast, colon, cervix, esophagus, pancreas, prostate, and lung.^2-8^

MISCAN Endometrial is a stochastic, semi-Markov, microsimulation model for endometrial cancer (EC) programmed in Delphi (Borland Software Corporation, Scotts Valley, California, United States). It can be used to explain and predict trends in EC incidence and mortality and to quantify the effects and costs of primary prevention of EC, screening for EC, and prophylactic hysterectomy. The term ‘microsimulation’ implies that the individuals are moved through the model one at a time, rather than as proportions of a cohort. The term ‘semi-Markov’ implies that MISCAN Endometrial, unlike traditional Markov models, does not assume annual state transitions; instead it generates durations in states, allowing future state transitions to depend on past transitions, and thereby increases model flexibility and computational performance. The term ‘stochastic’ implies that the model determines the states and corresponding durations by drawing from probability distributions, rather than using fixed values. Hence, the results of the model are subject to random variation.

The version of the MISCAN Endometrial model used for this manuscript consists of a demography module, a natural history module and a prophylactic hysterectomy module.

**Demography module**

Using birth- and life-tables, MISCAN Endometrial draws a date of birth and a date of non-EC death for each woman simulated. Birth tables were based on Leenen et al.,^9^ reflecting the age range of first-degree relatives of individuals diagnosed with lynch syndrome by universal testing of lynch syndrome in colorectal cancer. Life tables were based on National Vital Statistics Reports 2012,^10^ reflecting the life expectancy of women in the US. The maximum age an individual can achieve is assumed to be 100 years.

**Natural history module**

*Transitions*All women are born without lesions. As each simulated woman ages, a woman may develop endometrial hyperplasia, either atypical or without atypia (Figure 1). Hyperplasia without atypia was assumed to be 6.14 times more frequent than atypical hyperplasia.^11^ Hyperplasia may or may not progress into preclinical cancer, i.e cancer not yet giving symptoms. Over time, the preclinical cancer may start to give symptoms, resulting in cancer diagnosis. Upon diagnoses, women move to the clinical cancer state. After clinical diagnosis, EC survival is simulated using age-specific survival estimates based on 2009-2013 data from SEER 18.^12^ As it was assumed that women diagnosed with EC will receive hysterectomy combined with oophorectomy as part of their treatment, women can only develop endometrial hyperplasia and EC once during their lifetime. The date of death for individuals with EC is set to the earliest simulated death due either to EC or another cause (‘Demography module’).

**
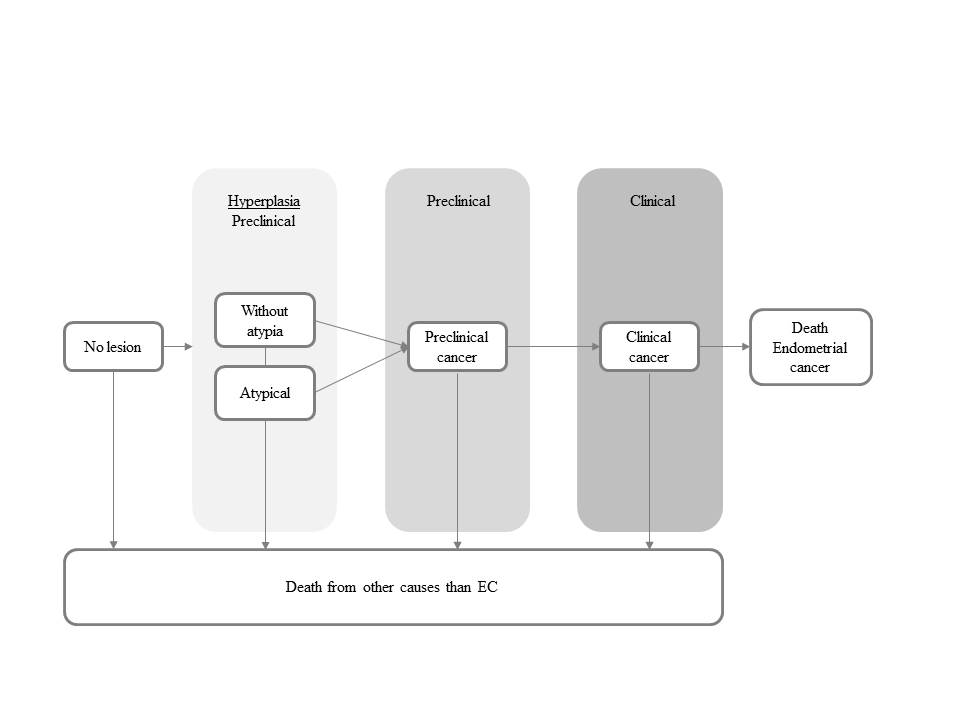
**

**Figure 1:** The stages of disease in the semi-Markov model.

*Transition rates and durations*A woman’s risk of developing hyperplasia depends on the woman’s age and a personal Gamma-distributed risk index (non-homogeneous Poisson process). The age-specific onset of hyperplasia was calibrated to the study by Bonadona et al,^13^ assuming a 35% risk of women diagnosed with lynch syndrome to develop EC before the age of 80. The progression of hyperplasia to endometrial cancer differed between hyperplasia without atypia and hyperplasia with atypia, since both have different dwelling times.^11^ Dwelling times were derived from Lacey et al. and were estimated with a Weibull distribution.^11^ In line with assumptions made for the development of colorectal cancer,^14^ preclinical lesions were assumed to progress 10 times faster in lynch syndrome patients than in the general population. Mean dwelling times for atypical hyperplasia and hyperplasia without atypia were 7.77 years and 114 years, respectively, reflecting that not all endometrial hyperplasia progresses to cancer.

**Figure 2:** Simulated versus observed endometrial cancer incidence, based on the study by Bonadona et al.^13^

**Prophylactic hysterectomy module**

In the prophylactic hysterectomy module, women of a certain age (e.g., 40-80 years) undergo prophylactic hysterectomy. Women who underwent a prophylactic hysterectomy are no longer at risk for developing endometrial hyperplasia and EC. If any preclinical hyperplasia was present at the time of the prophylactic hysterectomy, it is assumed that this is removed and that this no longer impacts a woman’s life expectancy.

**Integrating modules**

In Figure 3, the life history of an example patient is shown; this figure can be used to demonstrate how the different modules are integrated and how the benefit of prophylactic hysterectomy is quantified. For each individual simulated, the demography module first generates a date of birth and a date of non-EC death, creating a life-history without endometrial hyperplasia or EC. Then, the natural history module comes into play, generating onset of disease. For some women, the onset of disease falls after their age of death of other causes, and the woman will not develop any disease. In the example in Figure 3, the simulated woman develops atypical hyperplasia at a relatively young age. This hyperplasia progresses into preclinical cancer, which is diagnosed because of symptoms and results in EC death before non-EC death would have occurred. In the prophylactic hysterectomy module, a prophylactic hysterectomy is simulated, indicated by the blue arrow. In this example, the prophylactic hysterectomy is performed when the woman already developed atypical hyperplasia. As a consequence of the prophylactic hysterectomy, both EC and EC death are prevented. Hence, integrating all three modules, prophylactic hysterectomy prolongs life by the amount indicated by the green arrow.


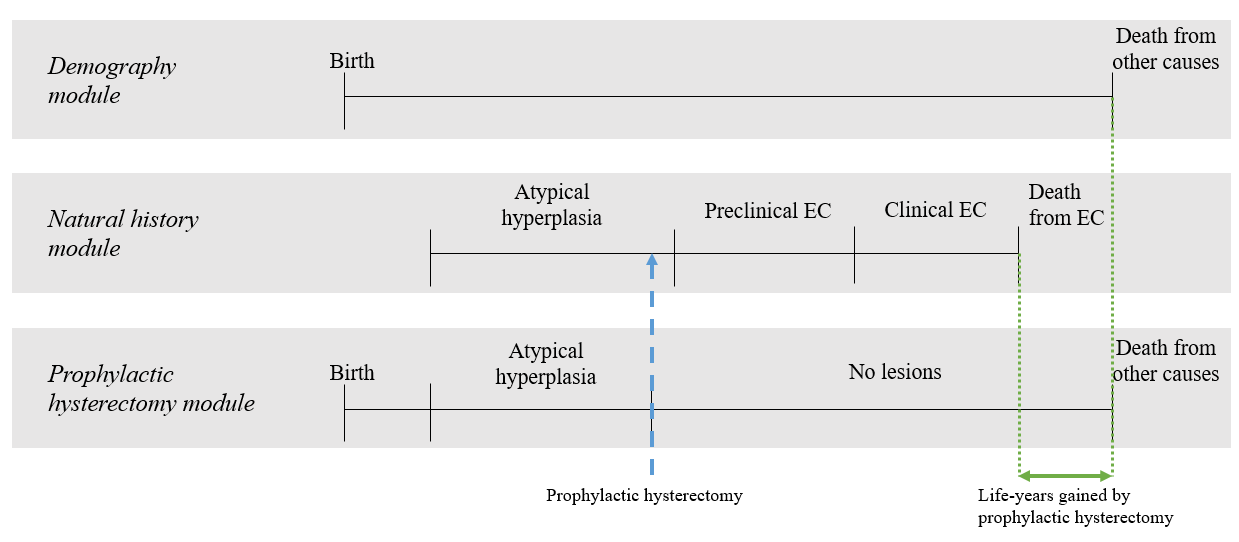
**Figure 3:** Integrating MISCAN modules for one example patient.

**References**

1. Habbema JD, van Oortmarssen GJ, Lubbe JT, van der Maas PJ. The MISCAN simulation program for the evaluation of screening for disease. *Comput Methods Programs Biomed.* 1985;20(1):79-93.

2. Draisma G, Etzioni R, Tsodikov A, et al. Lead time and overdiagnosis in prostate-specific antigen screening: importance of methods and context. *J Natl Cancer Inst.* 2009;101(6):374-383.

3. Loeve F, Boer R, van Oortmarssen GJ, van Ballegooijen M, Habbema JD. The MISCAN-COLON simulation model for the evaluation of colorectal cancer screening. *Comput Biomed Res.* 1999;32(1):13-33.

4. Ten Haaf K, de Koning HJ. Should Never-Smokers at Increased Risk for Lung Cancer Be Screened? *J Thorac Oncol.* 2015;10(9):1285-1291.

5. van den Akker-van Marle ME, van Ballegooijen M, van Oortmarssen GJ, Boer R, Habbema JD. Cost-effectiveness of cervical cancer screening: comparison of screening policies. *J Natl Cancer Inst.* 2002;94(3):193-204.

6. van der Maas PJ, de Koning HJ, van Ineveld BM, et al. The cost-effectiveness of breast cancer screening. *Int J Cancer.* 1989;43(6):1055-1060.

7. Kroep S, Lansdorp-Vogelaar I, Rubenstein JH, et al. An Accurate Cancer Incidence in Barrett's Esophagus: A Best Estimate Using Published Data and Modeling. *Gastroenterology.* 2015;149(3):577-585 e574; quiz e514-575.

8. Koopmann BDM, Harinck F, Kroep S, et al. Identifying key factors for the effectiveness of pancreatic cancer screening: A model-based analysis. *Int J Cancer.* 2021.

9. Leenen CH, Goverde A, de Bekker-Grob EW, et al. Cost-effectiveness of routine screening for Lynch syndrome in colorectal cancer patients up to 70 years of age. *Genet Med.* 2016;18(10):966-973.

10. Martin JA, Hamilton BE, Osterman MJ, Curtin SC, Matthews TJ. Births: final data for 2012. *Natl Vital Stat Rep.* 2013;62(9):1-68.

11. Lacey JV, Jr., Sherman ME, Rush BB, et al. Absolute risk of endometrial carcinoma during 20-year follow-up among women with endometrial hyperplasia. *J Clin Oncol.* 2010;28(5):788-792.

12. Surveillance, Epidemiology, and End Results (SEER) Program ([www.seer.cancer.gov](file:///\\storage.erasmusmc.nl\v\vcl13\MAGE\DATA\UserData\551009\02%20Manuscripts\Miscan%20-%20lynch\Manuscript%202020\www.seer.cancer.gov)) SEER*Stat Database: Incidence - SEER 18 Regs Research Data + Hurricane Katrina Impacted Louisiana Cases, Nov 2015 Sub (1973-2013 varying) - Linked To County Attributes - Total U.S., 1969-2014 Counties.

13. Bonadona V, Bonaiti B, Olschwang S, et al. Cancer risks associated with germline mutations in MLH1, MSH2, and MSH6 genes in Lynch syndrome. *JAMA.* 2011;305(22):2304-2310.

14. Peterse EFP, Naber SK, Daly C, et al. Cost-effectiveness of Active Identification and Subsequent Colonoscopy Surveillance of Lynch Syndrome Cases. *Clin Gastroenterol Hepatol.* 2019.
