# Supplementary material for: Cost‐effectiveness of prophylactic hysterectomy in first‐degree female relatives with Lynch syndrome of patients diagnosed with colorectal cancer in the United States: a microsimulation study
Source: Cancer Med. 2021 Sep 12;10(19):6835–44. doi: 10.1002/cam4.4080 (PMC8495276; doi:10.1002/cam4.4080)
Supplement: Supplementary file 2 — AppendixTables5‐14 [file CAM4-10-6835-s001.docx]

**Appendix**

**Table 5 : Results sensitivity analysis: -50% hysterectomy costs (per 1000 women diagnosed with Lynch syndrome)**

| Strategy | EC cases | EC deaths | LYG^a^ | QALYG^a^ | Costs^a,^ (million US$) | ACER QALYG^a,b^ | ICER QALYG |
| --- | --- | --- | --- | --- | --- | --- | --- |
| No prophylactic hysterectomy | 300 | 70.9 | **-** | **-** | 5.9 |  |  |
| 40-70 | 11.0 | 2.9 | 411 | 506 | 6.7 | $1,600 | $14,306 |
| 40-75 | 6.7 | 1.5 | 417 | 514 | 6.8 | $1,796 | $45,232 |
| 40-80 | 5.4 | 1.0 | 420 | 516 | 6.9 | $1,901 | $70,452 |
| 35-70 | 6.6 | 2.1 | 423 | 374 | 6.9 | $2,694 | Dominated |
|  |  |  |  |  |  |  |  |
| 35-75 | 2.3 | 2.9 | 430 | 381 | 7.0 | $2,935 | Dominated |
| 30-70 | 5.6 | 2.0 | 426 | 262 | 7.1 | $4,600 | Dominated |
| 35-80 | 1.0 | 0.2 | 432 | 384 | 7.1 | $3,070 | Dominated |
| 30-75 | 1.3 | 0.5 | 433 | 269 | 7.2 | $4,887 | Dominated |
| 30-80 | 0.0 | 0.0 | 435 | 272 | 7.3 | $5,061 | Dominated |

EC deaths endometrial cancer deaths, LYG life years gained, QALYG quality-adjusted life years gained, ACER Average Cost-Effectiveness Ratio
^a^ Results are 3% discounted
^b^ Compared to no prophylactic hysterectomy

| Strategy | EC cases | EC deaths | LYG^a^ | QALYG^a^ | Costs^a,^ (million US$) | ACER QALYG^a,b^ | ICER QALYG |
| --- | --- | --- | --- | --- | --- | --- | --- |
| No prophylactic hysterectomy | 300 | 70.9 | **-** | **-** | 5.9 |  |  |
| 40-70 | 11.0 | 2.9 | 411 | 506 | 26.0 | $39,718 | $39,718 |
| 40-75 | 6.7 | 1.5 | 417 | 514 | 26.8 | $40,714 | $106,335 |
| 35-70 | 6.6 | 2.1 | 423 | 374 | 27.2 | $56,817 | Dominated |
| 40-80 | 5.4 | 1.0 | 420 | 516 | 27.2 | $41,223 | $159,019 |
| 35-75 | 2.3 | 2.9 | 430 | 381 | 28.0 | $57,813 | Dominated |
| 30-70 | 5.6 | 2.0 | 426 | 262 | 28.0 | $84,461 | Dominated |
| 35-80 | 1.0 | 0.2 | 432 | 384 | 28.3 | $58,399 | Dominated |
| 30-75 | 1.3 | 0.5 | 433 | 269 | 28.8 | $85,081 | Dominated |
| 30-80 | 0.0 | 0.0 | 435 | 272 | 29.2 | $85,685 | Dominated |

**Table 6 : Results sensitivity analysis: +100% hysterectomy costs (per 1000 women diagnosed with Lynch syndrome)**

EC deaths endometrial cancer deaths, LYG life years gained, QALYG quality-adjusted life years gained, ACER Average Cost-Effectiveness Ratio
^a^ Results are 3% discounted
^b^ Compared to no prophylactic hysterectomy

| Strategy | EC cases | EC deaths | LYG^a^ | QALYG^a^ | Costs^a,^ (million US$) | ACER QALYG^a,b^ | ICER QALYG |
| --- | --- | --- | --- | --- | --- | --- | --- |
| No prophylactic hysterectomy | 300 | 70.9 | **-** | **-** | 5.9 |  |  |
| 40-70 | 11.0 | 2.9 | 411 | 530 | 13.2 | $8,988 | $8,988 |
| 40-75 | 6.7 | 1.5 | 417 | 541 | 13.5 | $9,296 | $32,470 |
| 40-80 | 5.4 | 1.0 | 420 | 375 | 13.7 | $9,453 | $52,237 |
| 35-70 | 6.6 | 2.1 | 423 | 544 | 13.7 | $11,348 | Dominated |
| 35-75 | 2.3 | 2.9 | 430 | 385 | 14.0 | $11,673 | Dominated |
| 30-70 | 5.6 | 2.0 | 426 | 245 | 14.1 | $14,251 | Dominated |
| 35-80 | 1.0 | 0.2 | 432 | 388 | 14.2 | $11,847 | Dominated |
| 30-75 | 1.3 | 0.5 | 433 | 255 | 14.4 | $14,584 | Dominated |
| 30-80 | 0.0 | 0.0 | 435 | 258 | 14.6 | $14,776 | Dominated |

**Table 7 : Results sensitivity analysis: utility endometrial cancer 0.68 (per 1000 women diagnosed with Lynch syndrome)**

EC deaths endometrial cancer deaths, LYG life years gained, QALYG quality-adjusted life years gained, ACER Average Cost-Effectiveness Ratio
^a^ Results are 3% discounted
^b^ Compared to no prophylactic hysterectomy

**Table 8 : Results sensitivity analysis: utility prophylactic hysterectomy 0.82 (per 1000 women diagnosed with Lynch syndrome)**

| Strategy | EC cases | EC deaths | LYG^a^ | QALYG^a^ | Costs^a,^ (million US$) | ACER QALYG^a,b^ | ICER QALYG |
| --- | --- | --- | --- | --- | --- | --- | --- |
| No prophylactic hysterectomy | 300 | 70.9 | **-** | **-** | 5.9 |  |  |
| 40-70 | 11.0 | 2.9 | 411 | 418 | 13.2 | $17,333 | $17,333 |
| 40-75 | 6.7 | 1.5 | 417 | 425 | 13.5 | $17,836 | $45,211 |
| 40-80 | 5.4 | 1.0 | 420 | 427 | 13.7 | $18,109 | $70,444 |
| 35-70 | 6.6 | 2.1 | 423 | 209 | 13.7 | $18,312 | Dominated |
| 35-75 | 2.3 | 2.9 | 430 | 217 | 14.0 | $18,835 | Dominated |
| 30-70 | 5.6 | 2.0 | 426 | 39 | 14.1 | $206,977 | Dominated |
| 35-80 | 1.0 | 0.2 | 432 | 219 | 14.2 | $37,692 | Dominated |
| 30-75 | 1.3 | 0.5 | 433 | 47 | 14.4 | $180,570 | Dominated |
| 30-80 | 0.0 | 0.0 | 435 | 49 | 14.6 | $175,617 | Dominated |

EC deaths endometrial cancer deaths, LYG life years gained, QALYG quality-adjusted life years gained, ACER Average Cost-Effectiveness Ratio
^a^ Results are 3% discounted
^b^ Compared to no prophylactic hysterectomy

**Table 9 : Results sensitivity analysis: utility prophylactic hysterectomy 0.99 (per 1000 women diagnosed with Lynch syndrome)**

| Strategy | EC cases | EC deaths | LYG^a^ | QALYG^a^ | Costs^a,^ (million US$) | ACER QALYG^a,b^ | ICER QALYG |
| --- | --- | --- | --- | --- | --- | --- | --- |
| No prophylactic hysterectomy | 300 | 70.9 | **-** | **-** | 5.9 |  |  |
| 40-70 | 11.0 | 2.9 | 411 | 668 | 13.2 | $10,837 | $10,837 |
| 40-75 | 6.7 | 1.5 | 417 | 675 | 13.5 | $11,228 | $45,727 |
| 40-80 | 5.4 | 1.0 | 420 | 678 | 13.7 | $11,422 | $70,467 |
| 35-70 | 6.6 | 2.1 | 423 | 676 | 13.7 | $11,470 | Dominated |
| 35-75 | 2.3 | 2.9 | 430 | 683 | 14.0 | $11,849 | $63,304 |
| 30-70 | 5.6 | 2.0 | 426 | 669 | 14.1 | $12,211 | Dominated |
| 35-80 | 1.0 | 0.2 | 432 | 686 | 14.2 | $12,039 | $70,467 |
| 30-75 | 1.3 | 0.5 | 433 | 677 | 14.4 | $12,585 | Dominated |
| 30-80 | 0.0 | 0.0 | 435 | 679 | 14.6 | $12,775 | Dominated |

EC deaths endometrial cancer deaths, LYG life years gained, QALYG quality-adjusted life years gained, ACER Average Cost-Effectiveness Ratio
^a^ Results are 3% discounted
^b^ Compared to no prophylactic hysterectomy

**Table 10: Results sensitivity analysis: risk endometrial cancer 17% (per 1000 women diagnosed with Lynch syndrome)**

| Strategy | EC cases | EC deaths | LYG^a^ | QALYG^a^ | Costs^a,^ (million US$) | ACER QALYG^a,b^ | ICER QALYG |
| --- | --- | --- | --- | --- | --- | --- | --- |
| No prophylactic hysterectomy | 284.0 | 69.0 | **-** | **-** | 5.4 |  |  |
| 40-70 | 6.3 | 2.2 | 363 | 416 | 13.1 | $18,569 | $18,569 |
| 40-75 | 1.8 | 0.6 | 370 | 425 | 13.5 | $19,036 | $42,351 |
| 40-80 | 0.4 | 0.1 | 372 | 427 | 13.6 | $19,308 | $70,927 |
| 35-70 | 6.0 | 2.1 | 363 | 269 | 13.7 | $30,833 | Dominated |
| 35-75 | 1.5 | 2.2 | 371 | 277 | 14.0 | $31,179 | Dominated |
| 30-70 | 5.9 | 2.1 | 364 | 157 | 14.1 | $55,556 | Dominated |
| 35-80 | 0.1 | 0.0 | 373 | 279 | 14.2 | $31,497 | Dominated |
| 30-75 | 1.4 | 0.5 | 371 | 165 | 14.5 | $54,885 | Dominated |
| 30-80 | 0.0 | 0.0 | 373 | 167 | 14.6 | $55,099 | Dominated |

EC deaths endometrial cancer deaths, LYG life years gained, QALYG quality-adjusted life years gained, ACER Average Cost-Effectiveness Ratio
^a^ Results are 3% discounted
^b^ Compared to no prophylactic hysterectomy

| Strategy | EC cases | EC deaths | LYG^a^ | QALYG^a^ | Costs^a,^ (million US$) | ACER QALYG^a,b^ | ICER QALYG |
| --- | --- | --- | --- | --- | --- | --- | --- |
| No prophylactic hysterectomy | 609.7 | 136.3 | **-** | **-** | 12.3 |  |  |
| 40-70 | 16.7 | 4.5 | 863 | 1344 | 13.4 | $787 | $787 |
| 40-75 | 9.9 | 2.2 | 875 | 1360 | 13.5 | $856 | $6,681 |
| 40-80 | 7.9 | 1.4 | 878 | 1363 | 13.6 | $892 | $13,572 |
| 35-70 | 10.2 | 3.3 | 882 | 1208 | 13.9 | $1,316 | Dominated |
| 35-75 | 3.5 | 4.5 | 893 | 1224 | 14.0 | $1,386 | Dominated |
| 35-80 | 1.5 | 0.2 | 897 | 1228 | 14.1 | $1,425 | Dominated |
| 30-70 | 8.8 | 3.1 | 886 | 1087 | 14.4 | 1,880 | Dominated |
|  |  |  |  |  |  |  |  |
| 30-75 | 2.0 | 0.8 | 897 | 1103 | 14.5 | $1,949 | Dominated |
| 30-80 | 0.0 | 0.0 | 900 | 1107 | 14.5 | $1,990 | Dominated |

**Table 11: Results sensitivity analysis: risk endometrial cancer 60% (per 1000 women diagnosed with Lynch syndrome)**

EC deaths endometrial cancer deaths, LYG life years gained, QALYG quality-adjusted life years gained, ACER Average Cost-Effectiveness Ratio
^a^ Results are 3% discounted
^b^ Compared to no prophylactic hysterectomy

| Strategy | EC cases | EC deaths | LYG^a^ | QALYG^a^ | Costs^a,^ (million US$) | ACER QALYG^a,b^ | ICER QALYG |
| --- | --- | --- | --- | --- | --- | --- | --- |
| No prophylactic hysterectomy | 297.5 | 69.9 | **-** | **-** | 5.9 |  |  |
| 40-70 | 10.9 | 2.9 | 404 | 495 | 13.2 | $14,670 | $14,670 |
| 40-75 | 6.7 | 1.5 | 411 | 503 | 13.5 | $15,132 | $45,244 |
| 40-80 | 5.4 | 1.0 | 413 | 505 | 13.7 | $15,372 | $70,569 |
| 35-70 | 6.5 | 2.1 | 417 | 363 | 13.7 | $21,445 | Dominated |
| 35-75 | 2.3 | 2.9 | 424 | 371 | 14.0 | $21,933 | Dominated |
| 30-70 | 5.5 | 1.9 | 420 | 251 | 14.1 | $32,748 | Dominated |
|  |  |  |  |  |  |  |  |
|  |  |  |  |  |  |  |  |
| 35-80 | 1.0 | 0.2 | 426 | 373 | 14.2 | $22,218 | Dominated |
|  |  |  |  |  |  |  |  |
| 30-75 | 1.3 | 0.5 | 426 | 258 | 14.4 | $33,115 | Dominated |
| 30-80 | 0.0 | 0.0 | 429 | 260 | 14.6 | $33,429 | Dominated |

**Table 12 : Results sensitivity analysis: Accounting for reduced life expectancy due to increased colorectal cancer risk in LS (per 1000 women diagnosed with Lynch syndrome)**

EC deaths endometrial cancer deaths, LYG life years gained, QALYG quality-adjusted life years gained, ACER Average Cost-Effectiveness Ratio
^a^ Results are 3% discounted
^b^ Compared to no prophylactic hysterectomy

**Table 13 : Results sensitivity analysis: -50% costs of treatment EC (per 1000 women diagnosed with Lynch syndrome)**

| Strategy | EC cases | EC deaths | LYG^a^ | QALYG^a^ | Costs^a,^ (million US$) | ACER QALYG^a,b^ | ICER QALYG |
| --- | --- | --- | --- | --- | --- | --- | --- |
| No prophylactic hysterectomy | 300 | 70.9 | **-** | **-** | 3.0 |  |  |
| 40-70 | 11.0 | 2.9 | 411 | 506 | 13.0 | $19,859 | $19,859 |
| 40-75 | 6.7 | 1.5 | 417 | 514 | 13.4 | $20,357 | $53,167 |
| 35-70 | 6.6 | 2.1 | 423 | 374 | 13.6 | $28,409 | Dominated |
| 40-80 | 5.4 | 1.0 | 420 | 516 | 13.6 | $20,611 | $79,509 |
| 35-75 | 2.3 | 2.9 | 430 | 381 | 14.0 | $28,906 | Dominated |
| 30-70 | 5.6 | 2.0 | 426 | 262 | 14.0 | $42,230 | Dominated |
| 35-80 | 1.0 | 0.2 | 432 | 384 | 14.2 | $29,199 | Dominated |
| 30-75 | 1.3 | 0.5 | 433 | 269 | 14.4 | $42,540 | Dominated |
| 30-80 | 0.0 | 0.0 | 435 | 272 | 14.6 | $42,842 | Dominated |

EC deaths endometrial cancer deaths, LYG life years gained, QALYG quality-adjusted life years gained, ACER Average Cost-Effectiveness Ratio
^a^ Results are 3% discounted
^b^ Compared to no prophylactic hysterectomy

**Table 14 : Results sensitivity analysis: +100% costs of treatment EC (per 1000 women diagnosed with Lynch syndrome)**

| Strategy | EC cases | EC deaths | LYG^a^ | QALYG^a^ | Costs^a,^ (million US$) | ACER QALYG^a,b^ | ICER QALYG |
| --- | --- | --- | --- | --- | --- | --- | --- |
| No prophylactic hysterectomy | 300 | 70.9 | **-** | **-** | 11.9 |  |  |
| 40-70 | 11.0 | 2.9 | 411 | 506 | 13.5 | $3,200 | $3,200 |
| 40-75 | 6.7 | 1.5 | 417 | 514 | 13.7 | $3,591 | $29,362 |
| 40-80 | 5.4 | 1.0 | 420 | 516 | 13.8 | $3,801 | $52,337 |
| 35-70 | 6.6 | 2.1 | 423 | 374 | 13.9 | $5,388 | Dominated |
|  |  |  |  |  |  |  |  |
|  |  |  |  |  |  |  |  |
| 35-75 | 2.3 | 2.9 | 430 | 381 | 14.1 | $5,870 | Dominated |
| 30-70 | 5.6 | 2.0 | 426 | 262 | 14.3 | $6,139 | Dominated |
| 35-80 | 1.0 | 0.2 | 432 | 384 | 14.3 | $9,200 | Dominated |
| 30-75 | 1.3 | 0.5 | 433 | 269 | 14.5 | $9,774 | Dominated |
| 30-80 | 0.0 | 0.0 | 435 | 272 | 14.6 | $10,122 | Dominated |

EC deaths endometrial cancer deaths, LYG life years gained, QALYG quality-adjusted life years gained, ACER Average Cost-Effectiveness Ratio
^a^ Results are 3% discounted
^b^ Compared to no prophylactic hysterectomy
